# Upregulated Seizure-Related 6 Homolog-Like 2 Is a Prognostic Predictor of Hepatocellular Carcinoma

**DOI:** 10.1155/2020/7318703

**Published:** 2020-02-13

**Authors:** Linhe Wang, Xiangchao Ling, Caihui Zhu, Zhiheng Zhang, Ziming Wang, Shanzhou Huang, Yunhua Tang, Shujiao He, Zhiyong Guo, Xiaoshun He

**Affiliations:** ^1^Organ Transplant Center, The First Affiliated Hospital, Sun Yat-sen University, Guangzhou 510080, China; ^2^Billary and Pancreatic Surgery, The First Affiliated Hospital, Sun Yat-sen University, Guangzhou 510080, China

## Abstract

Seizure-related 6 homolog-like 2 (*SEZ6L2*), which is localized on the cell surface, has been found to be associated with tumor angiogenesis and lung cancer progression. However, the role of *SEZ6L2* in hepatocellular carcinoma (HCC) is still unclear. We obtained data from The Cancer Genome Atlas (TCGA) and the Gene Expression Omnibus (GEO) to investigate *SEZ6L2* expression and regulation in HCC. Then, HCC tissue samples were collected to verify *SEZ6L2* by quantitative real-time polymerase chain reaction (qRT-PCR) and immunohistochemical staining (IHC). Patient information was collected for survival and prognosis analysis. qRT-PCR, IHC, and bioinformatics analysis showed that the SEZ6L2 protein was highly expressed in HCC samples. Clinical data showed that high SEZ6L2 protein expression was correlated with tumor-node-metastasis (TNM) stages (*P* = 0.046), tumor number (*P* = 0.016), and tumor size (*P* = 0.029). Meanwhile, SEZ6L2 overexpression was closely associated with poor overall survival and disease-free survival in HCC patients. Moreover, *SEZ6L2* is an independent prognostic predictor for the survival of HCC patients. This study suggests a significant correlation between *SEZ6L2* and HCC, which means that *SEZ6L2* may potentially serve as a useful prognostic biomarker for HCC patients.

## 1. Introduction

Hepatocellular carcinoma (HCC), a malignant cancer, is the most common primary liver cancer worldwide [1]. It is the third most common cause of cancer-related death and has been ranked second in cancer mortality in China since the 1990s [2, 3]. The major therapeutic methods used to treat HCC are hepatic resection, liver transplantation, chemotherapy, radiotherapy, and targeted agents [4]. Despite the apparent therapeutic effects of these treatment regimens, the rapid progression of the disease still leads to high mortality in patients [5, 6]. In recent studies, new immunotherapy and targeted therapies have achieved good results [7]. Therefore, it is rewarding to find effective gene markers for the early diagnosis and treatment of HCC.

Seizure-related 6 homolog-like 2 (*SEZ6L2*), which encodes a seizure-related protein localized on the cell surface, is located in the region of chromosome 16p11.2 and is predicted to be a candidate gene for autism spectrum disorders [8, 9]. This gene has broad expression in the brain, stomach, and testis and in the adrenal and prostate glands, while it is scarce in other normal tissues (from gene databases). Previous studies have shown that *SEZ6L2* regulates neurogenesis and neural differentiation and modulates *α*-amino-3-hydroxy-5-methyl-4-isoxazole propionic acid- (AMPA-) adducin (ADD) signal transduction through control of the phosphorylation of adducin [10, 11]. Moreover, a recent study found that AMPA-ADD is overexpressed in lung cancers and can be regarded as a significant prognostic biomarker for lung cancer [12]. However, the expression and clinicopathological significance of *SEZ6L2* in HCC remains unclear. Thus, this study aimed to detect the expression of *SEZ6L2* in HCC and determine the correlation between clinical value and the *SEZ6L2* expression level in HCC.

## 2. Materials and Methods

### 2.1. Patient Information and Clinical Tissue Samples

All tissue samples and clinical materials for this research, including 16 pairs of fresh-frozen HCC tissues and corresponding adjacent normal liver tissues (ANLT) and 95 formalin-fixed, paraffin-embedded HCC tissue samples, were from HCC patients after hepatic resection without radiotherapy and chemotherapy before surgery at the First Affiliated Hospital of Sun Yat-Sen University during the period from May 2012 to December 2014 [13]. All patients were followed up until June 2018. Information on the clinical characteristics of patients is shown in Table 1. All diagnoses were confirmed by pathology. This study was approved by the Ethics Committees of the First Affiliated Hospital of Sun Yat-Sen University in accordance with the 1964 Declaration of Helsinki (including later amendments) and relevant ethical standards. The clinical samples of patients were collected after obtaining written informed consent.

### 2.2. Quantitative Real-Time Polymerase Chain Reaction

A total of 16 pairs of RNA from HCC and ANLT were isolated from frozen tissues using RNA plus reagent (TaKaRa, Japan) according to the manufacturer's instructions. Complementary DNA was synthesized by a PrimerScript™ RT Reagent (TaKaRa, Japan) for mRNA according to the manufacturer's instruction. The *SEZ6L2* expression level was determined by qRT-PCR using a Power SYBR Green PCR Master Mix (Applied Biosystems). The sequence of genetic specific primers of *SEZ6L2* was as follows: *SEZ6L2* (forward: 5′-TTCACTGTGATTCGGGCTACC-3′, reverse: 5′-GGGGTTTCACCGTTCCAGG-3′) and GADPH (forward: TGTGGGCATCAATGGATTTGG, reverse: ACACCATGTATTCCGGGTCAAT) (Servicebio Technology, Wuhan, China). GADPH was used to standardize data as an endogenous control. PCR was performed as follows: 95°C for 30 seconds and then 40 cycles for 5 seconds in 95°C and 30 seconds in 60°C.

### 2.3. Immunohistochemical Staining (IHC)

Ninety-five samples of HCC tissue, which were paraffin-embedded, were sectioned in 4 *μ*m intervals. After deparaffinization, hydration, and blocking, the HCC tissue was incubated with a primary anti-SEZ6L2 antibody (Abcam, ab104194) overnight at 4°C. Then, the staining of HCC was observed under a microscope to evaluate the expression of the target SEZ6L2 protein. The results were analyzed and scored according to the percentage of positive cells. Staining intensity was scored as 0, 1, 2, and 3 for absent, weak, moderate, and strong, respectively. The percentage of positive results was defined as 0, 1, 2, and 3 for no positive results, less than 20%, 20% to 50%, and more than 50%, respectively. Each section was analyzed for a final score by adding the intensity of immunoreaction and the percentage of stained tumor cells and resulted in scores of 1 to 5. The scores of 0 and 1 were thought to have low expression, while the scores of 3, 4, and 5 were thought to have high expression.

### 2.4. Bioinformatics Analysis

The expression data of *SEZ6L2* in HCC was collected from the Gene Expression Profiling Interactive Analysis (GEPIA) online database (http://gepia.cancer-pku.cn/). The transcriptome profiling for gene expression and transcription factor analysis in HCC (407 tumor samples versus 58 normal samples) was looked up in The Cancer Genome Atlas (TCGA, http://gdc.cancer.gov/). The microarray data was downloaded from the Gene Expression Omnibus (GEO) datasets (GSE36376, GSE45436, and GSE6764) (http://www.ncbi.nlm.nih.gov/geo). The results were analyzed using Microsoft Excel 15.39, Cytoscape 3.6.1, R 3.6.1, and GraphPad Prism 7.0 software.

### 2.5. Statistical Analysis

Data were analyzed by SPSS version 20.0 (IBM, USA). The chi-squared test was used to demonstrate the relationship between *SEZ6L2* expression and other tumor characteristics. Kaplan-Meier and Cox proportional hazards were used to assess the effect of various prognostic factors on the overall survival (OS) and disease-free survival (DFS) of HCC patients. Differences were considered statistically significant when two tailed *P* < 0.05.

## 3. Results

### 3.1. SEZ6L2 Is Overexpressed in HCC Tissue by Transcription Factors such as GCNF

**Figure 1:**
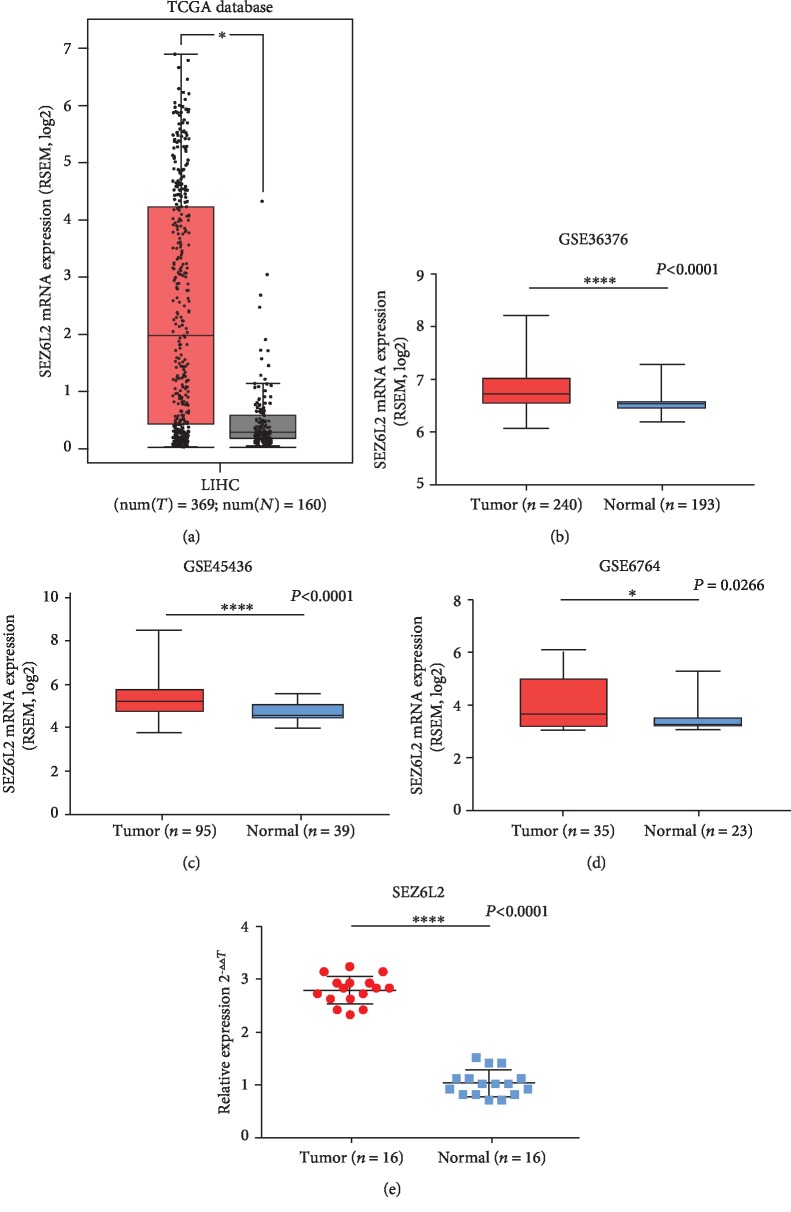
*SEZ6L2* is more expressed in HCC tissues than normal liver tissues according to the analysis of data from TCGA ((a) tumor, *n* = 369; normal, *n* = 160, ^∗∗∗∗^*P* < 0.0001) and GEO ((b–d) GSE36376: tumor (*n* = 240), normal (*n* = 193), ^∗∗∗∗^*P* < 0.0001; GSE45436: tumor (*n* = 95), normal (*n* = 39), *P* < 0.0001; GSE6764: tumor (*n* = 35), normal (*n* = 23), *P* = 0.0266). Real-time PCR analysis of relative *SEZ6L2* mRNA expression in 16 pairs of HCC tissues versus corresponding ANLT ((e) tumor, *n* = 16; normal, *n* = 16, ^∗∗∗∗^*P* < 0.0001). ∗ indicates a significant difference.

We downloaded the mRNA sequence or microarray data sets from TCGA and GEO, respectively, to examine the expression of *SEZ6L2* in HCC tissue. These data showed that *SEZ6L2* was obviously elevated in HCC tissue compared with normal tissues (Figures 1(a)–1(d)). Then, we analyzed the *SEZ6L2* expression level in 16 pairs of fresh-frozen HCC tissues and corresponding ANLT using qRT-PCR. The results revealed that *SEZ6L2* was upregulated in HCC tissue compared to nontumor sites (Figure 1(e)). Both of the outcomes demonstrated that *SEZ6L2* expression levels were significantly higher in HCC tissues versus corresponding ANLT.

**Figure 2:**
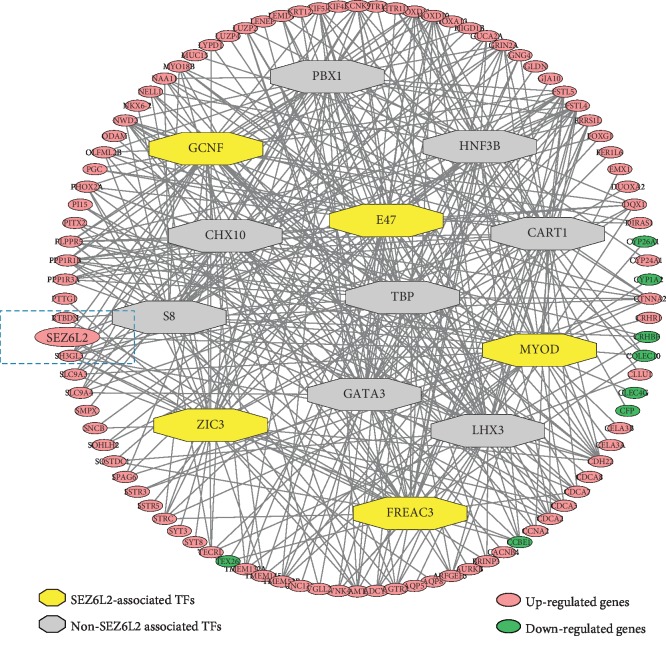
Transcription regulatory network analysis of HCC data from TCGA. The outer circles represent genes, red for upregulation and green for downregulation. The octagon in the middle of the circle represents transcription factors, and the yellow marks are transcription factors related to *SEZ6L2*. Line segments represent genes associated with transcription factors.

In order to explore the reasons leading to the high expression of *SEZ6L2* in HCC tissues, we analyzed 407 tumor samples versus 58 normal samples in HCC from the TCGA database. Then, we obtained the differentially expressed genes of these samples and made transcription regulatory network. Transcription factors GCNF, E47, MYOD, ZIC3, and FREAC3 were found to be associated with the upregulation of *SEZ6L2* (Figure 2).

### 3.2. SEZ6L2 Protein Expression Level Is Relevant in Clinicopathological Parameters of HCC

**Figure 3:**
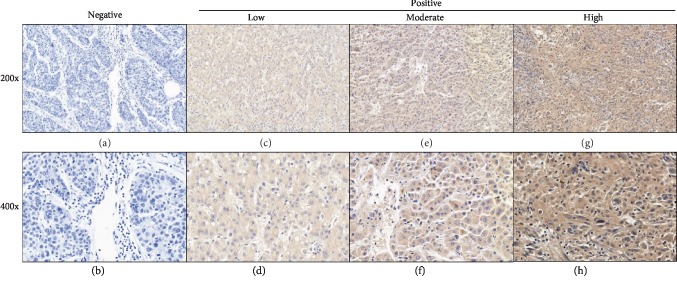
SEZ6L2 protein expression in 95 HCC tissues. Immunohistochemical staining showed negative (a, b), low (c, d), moderate (e, f), and high (g, h) stains for SEZ6L2 in HCC.

Ninety-five formalin-fixed, paraffin-embedded HCC tissue samples were used to detect SEZ6L2 protein expression using IHC in the search for the association between the SEZ6L2 protein expression and the clinicopathological parameters of HCC. Immunohistochemistry revealed that 31/95 cases (32.6%) had high intensity (>50% of tumor cells) of stain, 20/95 (21.1%) had moderate stain (20-50% of tumor cells), 29/95 (30.5%) had low (<20% of tumor cells) stain, and 15/95 (15.8%) of cases were negative (Figure 3).

Table 1 shows the relationship between SEZ6L2 protein expression and the clinicopathological parameters of HCC (low SEZ6L2 expression: negative and low positive group in IHC; high SEZ6L2 expression: high and moderate positive group in IHC). High SEZ6L2 protein expression levels were significantly correlated with tumor-node-metastasis stages (*P* = 0.046), tumor number (*P* = 0.016), and tumor size (*P* = 0.029), while there was no significant correlation with gender (*P* = 0.186), age (*P* = 0.612), or AFP (*P* = 0.984).

### 3.3. High SEZ6L2 Expression Is Associated with Poor Prognosis of HCC Patients

**Figure 4:**
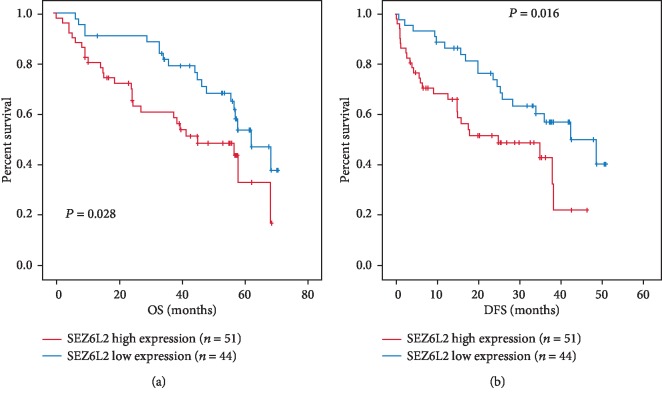
Overall survival (a) and disease-free survival (b) curves for the HCC patient groups with low (*n* = 44) and high (*n* = 51) SEZ6L2 expression.

Survival data was analyzed in 95 patients. The median OS time in HCC patients with low expression and high expression of SEZ6L2 was 62 months and 45 months, respectively, and showed a statistically significant difference (*P* = 0.028). The median DFS time in HCC patients with low expression and high expression of SEZ6L2 was 42 months and 25 months, respectively, and showed a statistically significant difference (*P* = 0.016). Kaplan-Meier analysis was used to calculate whether SEZ6L2 expression had prognostic value in HCC patients, and the curve showed that patients with high SEZ6L2 expression had worse OS and DFS times than patients with low SEZ6L2 expression (Figure 4).

Univariable Cox proportional-hazard results showed that TNM stage (hazard ratio (HR), 1.960; 95% confidence interval (CI) 1.088-3.533; *P* = 0.025), tumor number (HR, 0.547; 95% CI 0.277–1.081; *P* = 0.082), tumor size (HR, 0.534; 95% CI 0.290–0.983; *P* = 0.044), AFP (HR, 2.610; 95% CI 1.389–4.903; *P* = 0.003), and high SEZ6L2 expression (HR, 1.928; 95% CI 1.061–3.502; *P* = 0.031) were prognostic factors for OS (Table 2), while TNM stage (HR, 2.044; 95% CI 1.134–3.368; *P* = 0.017), tumor number (HR, 0.526; 95% CI 0.260–1.063; *P* = 0.074), tumor size (HR, 0.535; 95% CI 0.291–0.985; *P* = 0.045), AFP (HR, 2.890; 95% CI 1.540–5.423; *P* = 0.001), and high SEZ6L2 expression (HR, 2.083; 95% CI 1.135–3.825; *P* = 0.018) were prognostic factors for DFS (Table 3). Additionally, multivariable Cox proportional-hazard results showed that tumor size (HR, 0.461; 95% CI 0.235–0.905; *P* = 0.024), AFP (HR, 2.262; 95% CI 1.176–4.349; *P* = 0.014), and high SEZ6L2 expression (HR, 2.499; 95% CI 1.276–4.893; *P* = 0.008) were independent prognostic factors for OS (Table 2), while tumor size (HR, 0.435; 95% CI 0.221–0.855; *P* = 0.016), AFP (HR, 2.356; 95% CI 1.237–4.488; *P* = 0.009), and high SEZ6L2 expression (HR, 2.691; 95% CI 1.371–5.282; *P* = 0.004) were independent prognostic factors for DFS (Table 3). Therefore, high SEZ6L2 expression was correlated with OS as well as DFS and predicted poor survival in HCC patients.

## 4. Discussion

The *SEZ6L2* gene and the correlation between its expression level with its clinical value in HCC patients are still poorly understood. To our knowledge, this is the first report about the relationship between *SEZ6L2* expression and clinical prognostic survival in HCC patients. This study shows that *SEZ6L2* is overexpressed in HCC tissue, and its expression level is relevant in clinicopathological parameters of HCC. Moreover, high SEZ6L2 expression is associated with poor prognosis in HCC patients.

*SEZ6L2* is located in chromosome 16p11.2 and encodes a seizure-related protein that is localized on the cell surface. Previous studies have found that a coding variant in *SEZ6L2* has a significant association with autism spectrum disorders [9, 14]. Other research has demonstrated that SEZ6L2, a type I transmembrane protein and has Sushi/SCR and CUB domain outside the cellular surface, is highly expressed in the hippocampus and cerebellar cortex, and ataxia occurs in mice lacking members of the SEZ6L2 protein family [10]. Therefore, it can be affected by various drug delivery mechanisms. Many specific drugs such as Afatinib and Rituxan targets on cell surface proteins and have achieved the desired effect [15, 16]. Moreover, Hald and Galbo [17] found that *SEZ6L2* has extracellular epitopes, which are regarded as useful markers for the detection of pancreatic cell types at specific developmental stages by specific antibodies, and the Sushi and CUB domains are also involved in receptor-ligand interactions and cell adhesion. On the other hand, our results indicate that the SEZ6L2 protein is highly expressed in HCC tumor tissues, suggesting that it may be a potential therapeutic target. However, the role of *SEZ6L2* in HCC and its clinical and pathological significance is still unanswered.

In our study, the high expression of the *SEZ6L2* gene in HCC patients indicates that these patients may have a poor prognosis. Then, we searched for RNA sequencing data in TCGA (a public repository of comprehensive cancer genome maps) and obtained microarray data from GEO (a public repository that has high-throughput microarrays and next-generation sequential functional genome datasets) [18, 19]. The results revealed that *SEZ6L2* is upregulated in HCC tissue. And then, we investigated fresh-frozen HCC tissues using qRT-PCR compared with corresponding ANLT, which showed a similar consequence as that mentioned above.

According to previous research, *SEZ6L2* has been shown to be regulated by transcription factors, such as STAT3, which upregulate the expression of the vascular endothelial growth factor and promote tumor angiogenesis and cancer progression [20, 21]. So we did an analysis of the transcription regulatory network; our results indicated five transcription factors could upregulate the *SEZ6L2* gene, namely, GCNF, E47, MYOD, ZIC3, and FREAC3; no evidence shows that STAT3 can regulate the expression of *SEZ6L2* in HCC. However, the specific regulation mechanism of these transcription factors still needs further study.

To verify our results at the protein level, 95 formalin-fixed, paraffin-embedded HCC tissue samples were used to detect SEZ6L2 expression using IHC, which demonstrated that SEZ6L2 had high expression in HCC tissue. Finally, clinical data were collected for Kaplan-Meier analysis, as well as Cox proportional-hazard analysis, and we found that HCC patients with higher SEZ6L2 expression had lower OS and DFS, which means that *SEZ6L2* can act as a significant independent prognostic predictor for HCC patients.

This is the first article to prove that *SEZ6L2* is upregulated in HCC and can be a significant predictor for survival in HCC patients. A limitation of our research is that we did not study the molecular mechanisms of *SEZ6L2* function in HCC. Therefore, in the future, we intend to conduct more in-depth functional and mechanistic studies on mice and cell lines. In conclusion, our study demonstrates that *SEZ6L2* is overexpressed in HCC tissue and high SEZ6L2 expression is related to the poor prognosis of HCC. *SEZ6L2* can be considered a significant independent prognostic marker and potential therapeutic target for HCC.

## Figures and Tables

**Table 1:** Correlations between the SEZ6L2 expression and the clinicopathological variables of 95 HCC patients.

Variables	SEZ6L2 expression		*P*
	Low (*n* = 44)	High (*n* = 51)	
Sex			
Male	30	28	0.186
Female	14	23	
Age (y)			
<60	21	27	0.612
≥60	23	24	
AFP			
<400	23	27	0.984
≥400	21	24	
Tumor number			
Solitary	33	26	0.016
Multiple	11	25	
Tumor size			
≥2 cm	16	30	0.029
<2 cm	28	21	
TNM stages			
I & II	28	22	0.046
III & IV	16	29	

Abbreviations: HCC: hepatocellular carcinoma; AFP: alpha-fetoprotein; No.

**Table 2:** Cox proportional-hazard regression analysis for overall survival.

Variables	Univariable analysis			Multivariable analysis		
	HR	*P*	95% CI	HR	*P*	95% CI
Gender	1.252	0.452	0.697-2.250			
Age	1.313	0.359	0.734-2.349			
TNM stage	1.960	0.025	1.088-3.533	1.511	0.209	0.794-2.874
Cancer embolus	1.550	0.171	0.828-2.901			
HBV+HCV+	1.316	0.370	0.722-2.396			
Tumor number	0.547	0.082	0.277-1.081	0.590	0.171	0.277-1.256
Tumor size (≥2 cm)	0.534	0.044	0.290-0.983	0.461	0.024	0.235-0.905
AFP	2.610	0.003	1.389-4.903	2.262	0.014	1.176-4.349
Pathological grading	1.005	0.983	0.541-1.869			
SEZ6L2	1.928	0.031	1.061-3.502	2.499	0.008	1.276-4.893

Abbreviations: CI: confidence interval; HR: hazard ratio; AFP: alpha-fetoprotein; HBV: hepatitis B virus; HCV: hepatitis C virus.

**Table 3:** Cox proportional-hazard regression analysis for disease-free survival.

Variables	Univariable analysis			Multivariable analysis		
	HR	*P*	95% CI	HR	*P*	95% CI
Gender	1.313	0.361	0.732-2.356			
TNM stage	2.044	0.017	1.134-3.684	1.672	0.118	0.877-3.186
Cancer embolus	1.430	0.261	0.766-2.668			
HBV+HCV+	1.227	0.504	0.673-2.236			
Tumor number	0.526	0.074	0.260-1.063	0.545	0.104	0.262-1.133
Tumor size (≥52)	0.535	0.045	0.291-0.985	0.435	0.016	0.221-0.855
AFP	2.890	0.001	1.540-5.423	2.356	0.009	1.237-4.488
Pathological grading	0.956	0.888	0.514-1.780			
SEZ6L2	2.083	0.018	1.135-3.825	2.691	0.004	1.371-5.282

Abbreviations: CI: confidence interval; HR: hazard ratio; AFP: alpha-fetoprotein; HBV: hepatitis B virus; HCV: hepatitis C virus.

## Data Availability

The data used to support the findings of this study are available from the corresponding authors upon request.

## Abbreviations

*SEZ6L2*: Seizure-related 6 homolog-like 2
HCC: Hepatocellular carcinoma
TCGA: The Cancer Genome Atlas
GEO: Gene Expression Omnibus
qRT-PCR: Quantitative real-time polymerase chain reaction
IHC: Immunohistochemical staining
APMA: *α*-Amino-3-hydroxy-5-methyl-4-isoxazolepropionic acid
ADD: Adducin
PBS: Phosphate-buffered saline
OS: Overall survival
DFS: Disease-free survival
AFP: Alpha-fetoprotein
CI: Confidence interval
HR: Hazard ratio
ANLT: Adjacent normal liver tissues
GEPIA: Gene Expression Profiling Interactive Analysis.
